# Translation into Clinical Practice of the G1-G7 Molecular Subgroup Classification of Glioblastoma: Comprehensive Demographic and Molecular Pathway Profiling

**DOI:** 10.3390/cancers16020361

**Published:** 2024-01-15

**Authors:** Maria-Magdalena Georgescu

**Affiliations:** NeuroMarkers, Houston, TX 77025, USA; mmgeorgescu@yahoo.com

**Keywords:** glioblastoma, molecular classification, African American/Black, EGFR, NF1, PDGFRA, MET, PTEN, CDNK2A, CDK4, RB1

## Abstract

**Simple Summary:**

Glioblastoma is the most frequent and malignant primary tumor of the brain. Patient survival is dismal and personalized therapies are missing. In the diagnostic arena, the lack of an all-inclusive molecular subgroup classification for this tumor has delayed its personalized therapy. This study presents the validation of the first molecular subgroup classification of glioblastoma in a large patient cohort, and opens the avenue to rationalized therapeutic approaches. Preliminary comparative demographic studies between Caucasian/White and African American/Black patients offer the first comparative overview of the molecular diversity of glioblastoma. One molecular subgroup, G6/Multi-RTK, assembling several targetable gene fusions, is expanded and includes novel or rare examples of such events with associated tumorigenic mechanisms and cellular phenotypes. The bioinformatic analysis of the principal oncogenic pathways reveals important genetic associations and the extent of genomic heterogeneity at the populational level, offering the first organized molecular profiling of glioblastoma to be used as a foundation for therapy design, clinical practice and scientific research.

**Abstract:**

Glioblastoma is the most frequent and malignant primary neoplasm of the central nervous system. In a recent breakthrough study on a prospective Discovery cohort, I proposed the first all-inclusive molecular classification of glioblastoma into seven subgroups, G1-G7, based on MAPK pathway activation. New data from a WHO-grade-4 diffuse glioma prospective Validation cohort offers, in this study, an integrated demographic–molecular analysis of a 213-patient Combined cohort. Despite cohort differences in the median age and molecular subgroup distribution, all the prospectively-acquired cases from the Validation cohort mapped into one of the G1-G7 subgroups defined in the Discovery cohort. A younger age of onset, higher tumor mutation burden and expanded G1/EGFR-mutant and G3/NF1 glioblastoma subgroups characterized the glioblastomas from African American/Black relative to Caucasian/White patients. The three largest molecular subgroups were G1/EGFR, G3/NF1 and G7/Other. The fourth largest subgroup, G6/Multi-RTK, was detailed by describing a novel gene fusion *ST7–MET*, rare *PTPRZ1–MET*, *LMNA–NTRK1* and *GOPC–ROS1* fusions and their overexpression mechanisms in glioblastoma. The correlations between the MAPK pathway G1-G7 subgroups and the PI3-kinase/PTEN, TERT, cell cycle G1 phase and p53 pathways defined characteristic subgroup pathway profiles amenable to personalized targeted therapy. This analysis validated the first all-inclusive molecular classification of glioblastoma, showed significant demographic and molecular differences between subgroups, and provided the first ethnic molecular comparison of glioblastoma.

## 1. Introduction

Glioblastoma is the most frequent and deadly primary intraaxial neoplasm of the central nervous system (CNS) [1]. Its dismal prognosis is mainly due to diffuse brain invasion, tumor heterogeneity but also the activation of pathways that impart resistance to therapy. The World Health Organization (WHO) Classification of Central Nervous System Tumors assigns the highest tumor grade, WHO grade 4, to glioblastoma [2]. In addition, the most recent WHO classification reserves the terminology of isocitrate dehydrogenase (IDH)-wild-type glioblastoma only for the cases of WHO grade 4 diffuse gliomas without IDH or histone H3 mutations, henceforth called glioblastomas, which represent approximately 90% of all WHO grade 4 diffuse gliomas. The WHO grade 4 glioma cases showing IDH or histone H3 mutations are currently assigned to IDH-mutant astrocytoma, H3 K27-altered diffuse midline glioma (DMG) and H3 G34-mutant diffuse hemispheric glioma categories, respectively [2]. Noteworthy, the histologic derivation of all these four, currently distinct, WHO grade 4 gliomas is astrocytic, i.e., they are all high-grade astrocytomas, even if only one category, IDH-mutant astrocytoma, is named as such.

Risk stratification efforts for WHO grade 4 gliomas have been successful for the less common IDH-mutant astrocytoma tumors, for which the presence of *CDKN2A/2B* and *MYCN* alterations were correlated with significantly shorter survival [2,3]. For the large group of glioblastomas, there was no all-case-inclusive molecular classification until 2021, when I proposed the first integrated classification into seven molecular subgroups, G1-G7, showing distinct risk stratification [4]. Virtually all glioblastomas show the activation of the extracellular signal-regulated kinase/mitogen-activated protein kinase (ERK/MAPK) and phosphatidyl-inositol 3-OH kinase (PI3K) canonical growth pathways [4,5]. Noting non-redundant molecular alterations in the mediators of the ERK/MAPK pathway, including the receptor tyrosine kinases (RTKs) triggering pathway activation, I termed the seven molecular subgroups after the most upstream gene showing activating alterations: G1/EGFR, G2/FGFR3, G3/NF1, G4/RAF, G5/PDGFRA, G6/Multi-RTK, and G7/Other [4]. In the latter, the PI3K growth pathway is dominant.

The current study validates the proposed G1-G7 molecular classification of glioblastomas by assigning all new cases from a demographically distinct Validation patient cohort to the seven molecular subgroups proposed through the analysis of the initial Discovery patient cohort [4]. The Combined cohort assembling both Discovery and Validation cohorts contains demographic and molecular diversity, revealing important demographic–molecular correlations. A detailed analysis of the G6/Multi-RTK subgroup is provided and cases with novel or rare RTK fusions from this subgroup are exemplified. Most importantly, this is the first controlled study comparing the molecular assets of glioblastoma developing in Caucasian/White (C/W) and African American/Black (AA/B) patients.

## 2. Materials and Methods

*Validation cohort: tumor specimens*, *histology and immunohistochemistry (IHC).* The specimens form the Validation cohort were processed, examined and diagnosed similarly to those from the Discovery cohort [4]. Briefly, formalin-fixed paraffin-embedded (FFPE) sections from high-grade glioma biopsies or surgical resections were stained with hematoxylin and eosin (H&E) or various antibodies. All cases were tested for protein expression by IHC with antibodies for p53 (DO-7), Ki-67 (30-9) (Roche/Ventana Medical Systems Inc., Tucson, AZ, USA) and GFAP (EP672Y) (Ventana/Cell Marque, Rocklin, CA, USA), as described [6]. Low- and high-magnification images were acquired with Nikon Eclipse Ci microscope equipped with Nikon Digital Sight DS-Fi2 camera (Nikon Instruments Inc., Melville, NY, USA), as previously described [4]. An initial integrated histologic and molecular diagnosis, according to the most recent WHO guidelines [2], was obtained for all cases. The glioblastoma cases were further categorized into the G1-G7 molecular subgroups [4].

*Next-generation sequencing (NGS)*, *copy number variation (CNV) and transcriptomics.* The DNA and RNA NGS analyses were performed from FFPE samples using the xT-648-gene panel (Tempus Labs, Chicago, IL, USA), as previously described [7,8]. For each case, the same FFPE block was used for DNA and RNA extraction to allow for direct comparison of results. Variant and CNV assessment and interpretation, and the RNA expression analysis were performed as extensively described elsewhere [7,8].

*Western blot (WB) analysis.* Fresh frozen tumor tissue lysis and WB were performed as previously described [7,9]. The U251-MG glioblastoma cell line used for control has been described elsewhere [10,11]. Primary antibodies used were MET (D1C2), PDGFRα (D1E1E) and EGFR (D38B1) (Cell Signaling Technology, Danvers, MA, USA) and β-actin (Sigma-Aldrich, St. Louis, MO, USA).

*Statistical analysis.* Parametric unpaired *t*-test with or without Welch’s correction, nonparametric Mann–Whitney rank test and multiple variable correlation matrix analysis were performed using GraphPad Prism (Version 10.0.0, GraphPad Software, La Jolla, CA, USA). For pathway correlation matrix analysis, multiple linear regression tables were generated and used to calculate Pearson’s correlation coefficients by imputing the mutation information from 186 successfully sequenced glioblastoma cases. Confidence intervals for all these tests were 95%. Survival Kaplan–Meier curves were analyzed using log-rank (Mantel–Cox) and Gehan–Breslow–Wilcoxon tests for statistical significance, as described [4]. Statistical significance was considered as *p* < 0.05 for all tests. The percent accrual of cases in the molecular subgroups of the Validation cohort relative to the Discovery cohort was calculated using the formula 100 × (n_Vi_ − ñ_Di_)/ñ_Di_, where n_Vi_ represents the number of cases (n) per molecular subgroup (i) in the Validation (V) cohort, and ñ_Di_ is the normalized number of cases (ñ) per molecular subgroup (i) in the Discovery cohort (D) as follows: ñ_Di_ = n_Di_ × n_V_/n_D_, where n is the total number of cases from each cohort. Data were analyzed and plotted using Microsoft Excel (Version 16.54, Microsoft Corp., Redmond, WA, USA), and GraphPad Prism.

## 3. Results

### 3.1. Validation of the G1-G7 Subgroup Classification of Glioblastoma

**Figure 1:**
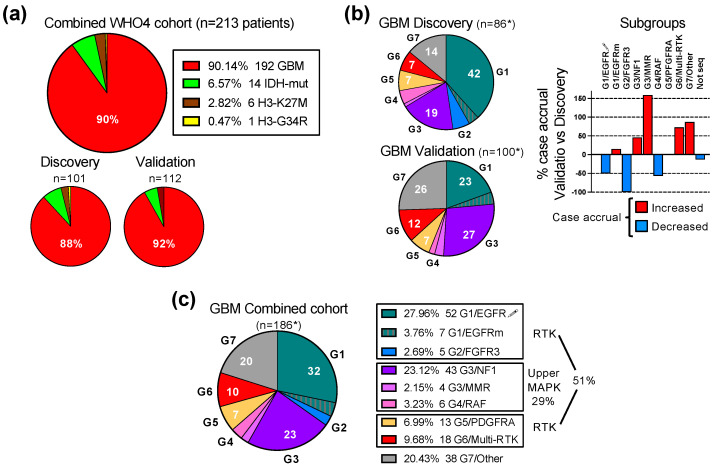
Validation of the G1-G7 molecular subgroup classification of glioblastoma. (**a**) Pie chart distribution (%) of WHO grade 4 glioma (WHO4) cases from the Combined patient cohort assembled from the initial Discovery cohort [4] and subsequent Validation cohort; GBM, glioblastoma; IDH-mut, astrocytoma IDH-mutant; H3-K27M, DMG H3 K27M-mutant; H3-G34R, diffuse hemispheric glioma H3 G34-mutant. (**b**) Left: pie chart distribution (%) of GBM cases in color-coded G1-G7 molecular subgroups in the Discovery and Validation cohorts. The asterisk (*) indicates total number of cases with NGS results, and does not include 3 cases from each cohort with failed NGS. The subgroup color is specified in (**c**) legend. The numbers from the pie charts indicate the % of cases in the respective molecular subgroup. Right: bar graph showing the relative case accrual in the Validation cohort in comparison to the number of cases from the initial Discovery cohort. Red and blue bars represent relative case increases or decreases, respectively. (**c**) Pie chart distribution (%) of GBM cases in color-coded G1-G7 molecular subgroups in the Combined cohort. Notations as in (**b**) above.

The study establishing the first unifying molecular classification of IDH-wild-type glioblastoma into seven molecular subgroups based on the activation of the ERK/MAPK pathway was performed on a prospective Discovery cohort of 101 adult patients with WHO grade 4 gliomas, of which 89 presented with IDH-wild-type glioblastoma [4]. Subsequently, a prospective Validation cohort containing 112 cases of WHO grade 4 diffuse gliomas, including 103 glioblastomas, was assembled (Figure 1a). Overall, the cases were derived mainly from three clinical centers located in the Southern United States. Both the Prospective and Validation cohort accrual was based on the same requirements: (1) prospective analysis; (2) all-inclusive accrual, i.e., no case was skipped or considered an outlier; (3) a WHO glioma diagnosis based on the most recent WHO recommendations; (4) molecular analysis consisting of both DNA and RNA NGS attempted for all cases. The Validation and Discovery cohorts showed a very similar overall composition, with an approximately 90% glioblastoma preponderance. The Combined cohort formed by joining the Discovery and Validation cohorts contained 213 WHO grade 4 gliomas, including 192 glioblastomas and 21 IDH-mutant and histone H3-mutant WHO grade 4 cases, the latter two at a 2:1 ratio, respectively (Figure 1a).

The NGS was successful for 86 and 100 glioblastoma cases in the Discovery and Validation cohorts, respectively (Figure 1b). For six cases, equally divided between the Discovery and Validation cohorts, NGS was not successful. The prospective inclusion of cases into the seven molecular subgroups showed a different distribution in the cohorts, with an overexpanded G1/EGFR subgroup in the Discovery cohort, and a balanced partition between the three major subgroups G1/EGFR, G3/NF1 and G7/Other in the Validation cohort (Figure 1b). Despite the variable case accrual per subgroup in the Validation cohort compared to the Discovery cohort (Figure 1b, graph), all of the glioblastoma cases accrued in the Validation cohort could be subclassified in one of the seven molecular subgroups, thus validating the G1-G7 molecular classification of glioblastoma.

Regardless of the case distribution in the cohorts, the three major subgroups combined, G1/EGFR, G3/NF1 and G7/Other, summed over 75% of the cases (Figure 1b,c). The four RTK subgroups, G1/EGFR, G2/FGFR3, G5/PDGFRA and G6/Multi-RTK, totaled over 50% of the cases, underscoring the importance of RTK signaling for glioblastoma pathogenesis. The G1/EGFR subgroup, composed of a majority of cases with EGFR amplification and overexpression (EGFR-amplified), and few cases with EGFR mutation without overexpression (EGFR-mutant), represented the largest RTK subgroup in the Combined cohort, due to the large number of EGFR-amplified cases contributed by the Discovery cohort [4].

Interestingly, the Validation cohort, despite a more balanced case distribution in molecular subgroups, did not contribute any cases to the G2/FGFR3 subgroup (Figure 1b graph). The cases in this minor subgroup show mainly constitutively activating FGFR3 gene fusions that are detected by RNA sequencing [7,12]. If RNA NGS is not performed or is not successful, false negative FGFR3 cases may, by exclusion, only map to the G7/Other subgroup. The examination of the G7/Other subgroup from the Validation cohort showed two cases for which limited tumor material was used only for DNA NGS. These cases showed *CDK4* amplification and high-grade neuroendocrine morphology that are commonly seen in the G7/Other subgroup but are not reported for the G2/FGFR3 subgroup [4,7,13], supporting that the Validation cohort lacked G2/FGFR3 cases.

Over a quarter of the glioblastoma cases mapped to the G3/NF1 and G4/RAF subgroups with oncogenic mutations in the upper segment of the ERK/MAPK signaling cascade (Figure 1c). Four cases with germline mutations in the mismatch repair (MMR) genes were assigned to their own G3/MMR subgroup, as they frequently show RAS-activating events, in addition to a characteristically high tumor mutation burden (TMB).

The G7/Other represents the subgroup of glioblastomas without RTK or upper ERK/MAPK segment activating alterations, and relies on the PI3K pathway for canonical growth signaling. A fifth to a quarter of glioblastoma cases will fall within this subgroup, and RNA sequencing is necessary for the correct classification and exclusion of the cases with RTK or upper ERK/MAPK segment fusions from this subgroup (Figure 1b,c).

### 3.2. Molecular–Demographic Correlations in Glioblastoma

**Figure 2:**
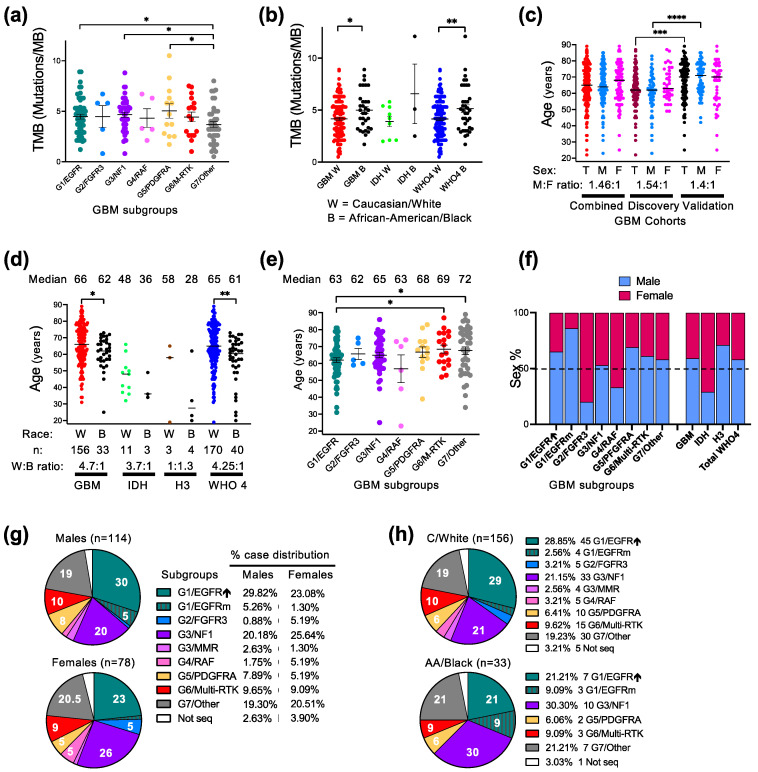
Demographic–molecular correlations. (**a**,**b**) Tumor mutation burden (TMB) mean ± SEM graph of individual case values from GBM molecular subgroups (**a**) or from C/W and AA/B patients with GBM, IDH-mutant astrocytoma (IDH), or combined WHO grade 4 (WHO4) gliomas (**b**). G3/MMR cases are not included in this analysis due to very high TMB (Table 2). Statistical significance: *, *p* < 0.05; **, *p* < 0.01. (**c**) Age distribution showing median of individual values in male (M), female (F) or total male and female (T) patients with GBM. Statistical significance: ***, *p* < 0.001; ****, *p* < 0.0001. (**d**) Age distribution with median indicated in C/W and AA/B patients with GBM, IDH-mutant, histone H3-mutant (H3) or WHO4 gliomas. Statistical significance (*) as in (**b**). (**e**) Age distribution in glioblastoma molecular subgroups as mean ± SEM. The median age is indicated on top of each subgroup. Statistical significance (*, **) as in (**b**). (**f**) Bar graph indicating the % of male and female distribution in GBM molecular subgroups, as well as in the glioma categories specified in (**d**). (**g**,**h**). Pie chart distribution (%) of cases from male/female patients (**g**) or C/W and AA/B patients (**h**) into the G1-G7 GBM molecular subgroups.

To better characterize the relation between the molecular spectrum and demographic characteristics in glioblastoma, several molecular and demographic parameters were examined (Table 1 and Table 2). As previously reported [4], the glioblastoma subgroups showed a TMB in the low range, the cut-off being 10 mutations/megabase. The G7/Other subgroup showed a lower TBM than the other subgroups, with statistical significance versus the G1/EGFR, G3/NF1 and G5/PDGFRA subgroups (Figure 2a). TMB comparison between the C/W and AA/B populations showed a significantly higher TMB in the AA/B tumors (Figure 2b).

The mean glioblastoma onset age, recorded as the date of the first surgery, differed significantly between the cohorts, showing 8 years of difference (Table 1 and Figure 2c). The Validation cohort contained an older population of both sexes, with the male population significantly older than the one in the Discovery cohort (Figure 2c). The Combined cohort showed a median onset age of 65 years, which is comparable with the age reported by WHO [2]. The male-to-female ratio was also similar in the Discovery and Validation cohorts, with a male preponderance (Table 1). The race composition was also similar between the two cohorts, with a 4.7:1 C/W to AA/B ratio in the Combined cohort (Table 1 and Supplementary Figure S1). The significant difference in age between the cohorts concerned only the C/W population (Supplementary Figure S1). The resulting Combined cohort showed a significant difference of the glioblastoma onset age between the C/W and AA/B populations (Figure 2d). This difference was even more appreciable in the larger Combined WHO grade 4 glioma cohort, due to the overrepresentation of AA/B patients in the IDH-mutant and histone H3-mutant WHO grade 4 diffuse gliomas as compared to glioblastoma (Figure 2d).

The molecular subgroup demographic analysis for the Combined glioblastoma cohort found a significantly higher patient age for the G7/Other and G6/Multi-RTK subgroups versus the G1/EGFR subgroup (Table 2 and Figure 2e). This difference coupled to the significant difference in the onset age between the cohorts may explain the reverse subgroup distribution in the two cohorts between the G1/EGFR, on one hand, and G7/Other and G6/Multi-RTK, on the other hand (Figure 1b).

Of the three major subgroups, the G1/EGFR subgroup showed a male overrepresentation regardless of race, with a very high preponderance of AA/B males in the G1/EGFR subgroup (Table 2). The AA/B males were also slightly preponderant in the G3/NF1 subgroup, but the C/W female and males were balanced, resulting in a balanced overall sex distribution in this subgroup (Table 2 and Figure 2f). The G7/Other subgroup showed a male overrepresentation for the C/W population but slight predominance of AA/B females over males, resulting in a milder overall male preponderance in this major subgroup. In the whole cohort, except for the G3/NF1 subgroup, the sex distribution was skewed, with a male to female predominance in the G1/EGFR, G5/PDGFRA, G6/Multi-RTK and G7/Other subgroups, and inversed ratio in the smaller subgroups G2/FGFR3 and G4/RAF (Figure 2f).

In general, the case distribution in both the major and minor subgroups appeared balanced for the female patients, whereas the male patients showed a skewed distribution in the G1/EGFR subgroup (Figure 2g). Compared to the C/W case distribution, the tumors from the AA/B patients predominated in the G1/EGFR-mutant and G3/NF1 subgroups, whereas the small subgroups G2/FGFR3, G3/MMR and G4/RAF did not contain any cases (Figure 2h).

### 3.3. Characterization of the G6/Multi-RTK Subgroup of Glioblastoma

**Figure 3:**
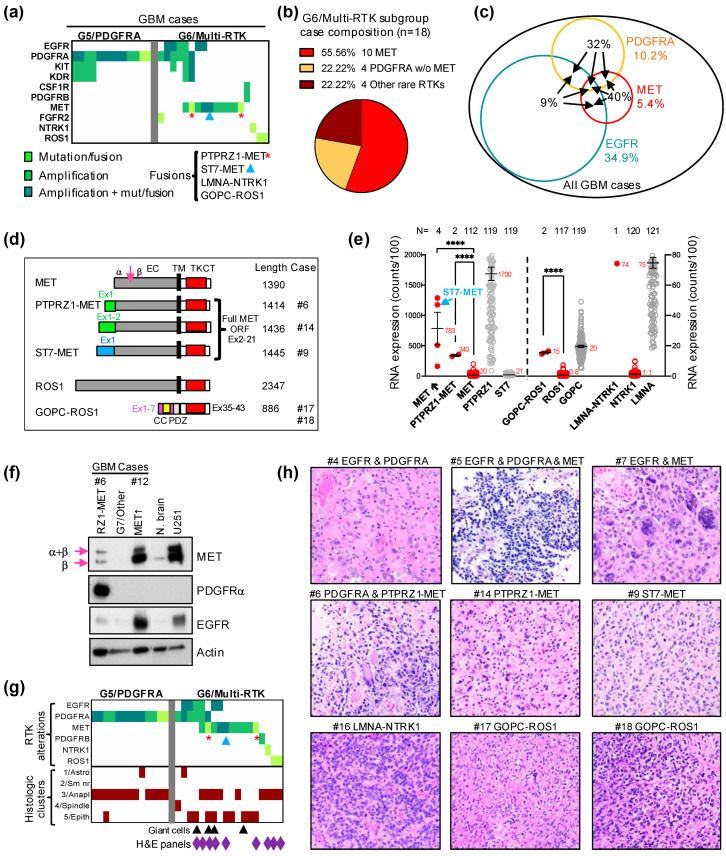
G6/Multi-RTK molecular subgroup characterization. (**a**). Matrix representation of the individual GBM cases from the G5/PDGFRA and G6/Multi-RTK molecular subgroups. (**b**). Pie chart composition of G6/Multi-RTK cases; *w*/*o*, without. (**c**). Venn diagram showing the % of GBM cases with *EGFR* amplifications, *PDGFRA* and *MET* alterations by colored numbers. The black number inside the circles represents the % of the respective RTK cases involved in associations (arrows) with other RTKs. (**d**). Schematic drawing with indicated amino acid lengths of the *MET* and *ROS1* fusions. Domains: EC, extracellular; TM, transmembrane; TK, tyrosine kinase; CT, carboxyl terminus; CC, coiled coil; PDZ, PSD95-DLG1-ZO1. MET RTK is cleaved (arrow) into two chains, a and b, that are linked via disulfide bridges to form the mature receptor. (**e**). Double *Y*-axis mean ± SEM graph of mRNA expression from individual cases with *MET* amplification (↑), *PTPRZ1–MET*, *GOPC–ROS1* and *LMNA–NTRK1* fusions. Control *MET*, *PTPRZ1*, *ST7*, *ROS1*, *GOPC*, *NTRK1* and *LMNA* from the cohort are shown for comparison. The number of cases is indicated on top, and the mean expression value is indicated by a red number next to the mean bar. *ST7–MET* fusion is amplified and its value is indicated by blue arrow. Statistically significant differences are shown: ****, *p* < 0.0001. (**f**). WB with indicated antibodies for G6/Multi-RTK cases #6 with *PTPRZ1–MET* (RZ1–MET) fusion and #12 with *MET* amplification (↑), and a G7/Other case for comparison. N. brain, normal autopsy brain tissue lysate and U251, U251-MG glioblastoma cell line lysate, are shown as controls. The immature (α + β) and mature β chains of MET are indicated by arrows. (**g**). Matrix representation of individual cases as in (**a**) with the corresponding histologic clusters of the tumors. Tumors showing giant cells are indicated by black arrowheads. The cases with illustrated morphology in (**h**) are indicated by purple diamonds. (**h**). H&E panels showing tumor morphology for the indicated cases at 200× magnification; high-resolution images are shown in Supplementary Figure S2.

The G5/PDGFRA and G6/Multi-RTK are minor molecular subgroups, composing less than one fifth of all glioblastoma cases (Figure 1c). The G6/Multi-RTK subgroup tripled its size in the Combined cohort by accruing a relatively high number of cases in the Validation cohort (Figure 1b, graph), thus becoming the fourth largest molecular glioblastoma subgroup (Figure 1c). Initially, many cases from the G6/Multi-RTK subgroup contained *PDGFRA* amplification in addition to an activating alteration in another RTK [4]. The initial observation that the *PDGFRA* expression was blunted for the cases with *PDGFRA* amplification from the G6/Multi-RTK subgroup was not confirmed for all the newly accrued cases. Therefore, simplified genomic criteria were adopted for the inclusion of cases with a *PDGFRA* amplification in these subgroups. Thus, the G5/PDGFRA subgroup contains tumors with an amplification of *PDGFRA* with or without neighboring *KIT* and *KDR* RTK genes from the 4q12 chromosomal locus, and more rarely, a tumor with *PDGFRA* activating mutations without amplification (Figure 3a). The G6/Multi-RTK subgroup assembles (1) tumors with *MET* alterations, of which 40% also occurred in combination with other RTK alterations; all these MET tumors represented 5.4% of the Combined cohort; (2) tumors with *PDGFRA* amplification in combination with other RTK alterations; and (3) cases with less common RTK alterations, such as in *NTRK1*, *ROS1* and *PDGFRB* (Figure 3a–c). Although 40% of the MET-altered cases occurred in combination with either *PDGFRA* or *EGFR* amplifications, the highest number of cases co-altered involved either *EGFR* or *PDGFRA* amplifications, six of each in the G6/Multi-RTK subgroup, and representing 9% and 32% of the total EGFR or PDGFRA cases from the Combined cohort, respectively (Figure 3a,c). The co-existence of amplifications in multiple RTKs in the same tumor has been previously described by FISH and array CGH (comparative genomic hybridization) for combinations of *PDGFRA* and *EGFR* or *MET*, *EGFR* and *MET*, and also one case suggested with triple amplification [14,15,16]. Moreover, in these reports, *PDGFRA* and *EGFR* amplifications usually occurred in distinct tumor cell populations and rarely simultaneously in the same tumor cells, although a common parental origin was postulated based on mutation analysis [15,16]. In the G6/Multi-RTK subgroup from the Combined cohort, all of these combinations were encountered, except that the combination of the *PDGFRA* and *MET* amplifications was replaced by *PDGFRA* amplification and *PTPRZ1-MET* activating fusion (Figure 3a).

*MET* activation resulted from the amplification of the *MET* locus on chromosome 7q31.2, *MET* fusions, or a combination of these. A likely pathogenic mutation in the MET extracellular domain, p.G764W, was detected in one case with amplified *MET*. Two *PTPRZ1*–*MET* fusions without amplification, and a novel fusion, *ST7–MET*, with amplification, were also detected (Figure 3a,d). *PTPRZ1–MET* fusions connecting the first one, two, three or eight exons of the receptor tyrosine phosphatase *PTPRZ1* with exon 2 of *MET* just before the open reading frame (ORF) start codon have been previously reported in glioblastoma [17] (Figure 3d). The *ST7–MET* fusion that couples the first exon of ST7 (suppressor of tumorigenicity 7) is a novel finding in glioblastoma, and has been described as an oncogenic event in non-small-cell lung carcinoma [18] (Figure 3d). Interestingly, whereas the *ST7–MET* fusion co-occurred with an *MET* amplification and showed overexpression levels similar to other *MET*-amplified cases, the *PTPRZ1–MET* fusions occurred in the absence of amplification, but showed significant *MET* overexpression (Figure 3a,e). The mRNA expression analysis also showed that the basal expression of both *MET* and *ST7* are very low in the glioblastoma cases, in contrast to the very high basal expression of *PTPRZ1* (Figure 3e). This suggested that the promoter of *PTPRZ1* is sufficient to induce the significant overexpression of the fusion product, whereas the *ST7–MET* fusion is overexpressed by simultaneous *MET/ST7* locus amplification (Figure 3e). Consistent with these mRNA expression results, the WB analysis of two *MET*-altered cases from the G6/Multi-RTK subgroup showed very high MET expression for the *MET*-amplified case #12, and a high MET expression for the *PTPRZ1–MET* fusion case #6 that also showed very high PDGFRα expression due to simultaneous *PDGFRA* amplification (Figure 3a,f). For comparison, lysates from a G7/Other case without any RTK alterations, normal brain [7] and U251-MG glioblastoma cell line [11] were run in parallel (Figure 3f). This analysis also showed that the maturation of the PTPRZ1–MET protein to the β chain in the tumor samples is similar to that of amplified MET, if not less efficient. The slight increase in the protein length of 24 amino acids in PTPRZ1–MET relative to MET (Figure 3d) cannot be visualized on WB, and leaky ribosome scanning to the *MET* start codon is also a possibility for both the shorter *PTPRZ1–MET* fusion forms and for *ST7–MET*. 

Rare *LMNA–NTRK1* and *GOPC–ROS1* fusions have been previously reported in glioblastoma [19,20], and their incidence in the Combined cohort is very low, at 0.5% and 1.1%, respectively (Figure 3a). Similar to the *PTPRZ1*–*MET* fusions, the *GOPC–ROS1* fusions resulted in an approximately 20-fold overexpression of *ROS1* in the absence of *ROS1* amplification, most likely driven by the *GOPC* (Golgi-associated PDZ and coiled-coil motif containing) promoter that showed also comparable expression to the fusion product (Figure 3e). However, this overexpression appears to represent just one mechanism of activation, as the GOPC–ROS1 protein has been shown to also be constitutively activated by mislocalization to the Golgi apparatus [21]. Similarly, the *LMNA–NTRK1* fusion showed an over 70-fold overexpression of *NTRK1*, comparable to the high *LMNA* expression from glioblastoma.

The morphologic classification of glioblastoma in 12 patterns assigned to five histologic clusters has been previously described [4]. The examination of the new cases from the Validation cohort confirmed the relatively homogenous appearance of the G5/PDGFRA subgroup tumors, in which almost all cases showed pre-high-grade neuroendocrine (HGNE) or HGNE histologic patterns, included in the #3/Anaplastic cluster (Figure 3g). Although the initial evaluation of the few G6/Multi-RTK subgroup tumors appeared more heterogenous [4], the majority of G6/Multi-RTK cases from the Combined cohort showed morphological profiles mapping to the clusters #3/Anaplastic, #5/Epithelioid or both (Figure 3g,h; Supplementary Figure S2). The #5/Epithelioid cluster morphological profiles were most often gemistocytic, including the cases with *ST7–MET* and *PTPRZ1–MET* fusions, and some showed giant cells (Figure 3g, black arrowheads, Figure 3h). Most of the cases classified in the #3/Anaplastic cluster showed pre-HGNE morphology, including the cases with *LMNA–NTRK1* and *GOPC–ROS1* fusions, with only the two cases with concurrent *PDGFRA* and *MET* alterations showing HGNE morphology (Figure 3h, Supplementary Figure S2, cases #5 and #6).

### 3.4. The Landscape of the Major Oncogenic Pathways in the G1-G7 Molecular Subgroups

**Figure 4:**
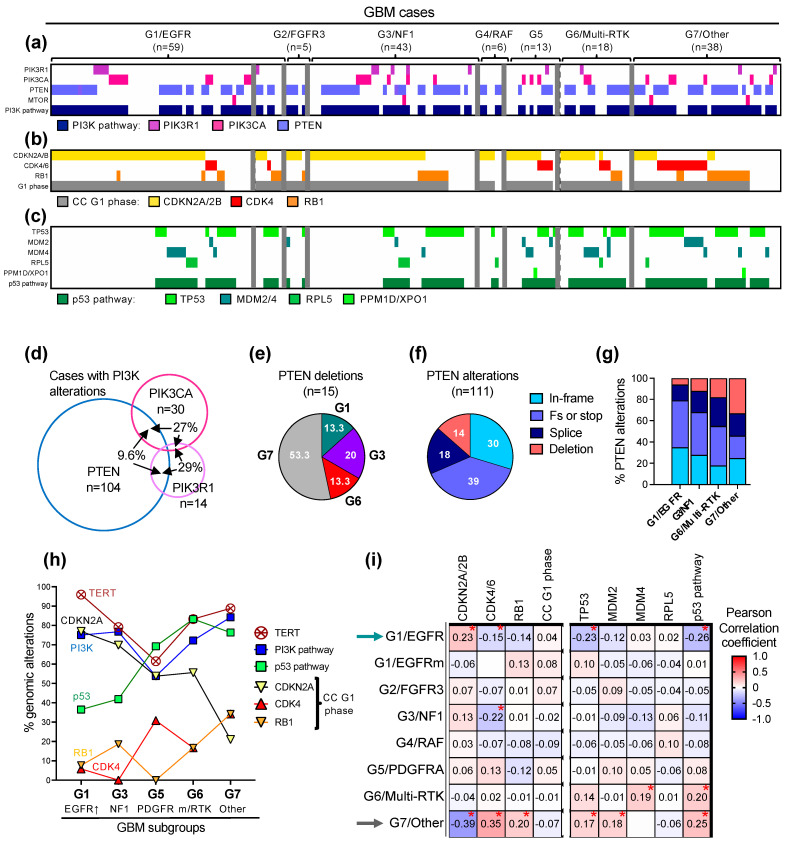
Profiling of the major growth pathways in the G1-G7 molecular subgroups. (**a**–**c**) Genomic alterations in effectors of the PI3K pathway (**a**), cell cycle G1 phase (**b**) and p53 pathway (**c**) are indicated by color-coded bars. The G1/EGFR subgroup cases are separated in EGFR-amplified (first 52 cases) and EGFR-mutant (last 7 cases). (**d**) Venn diagram showing the cases with overlapping *PTEN*, *PIK3CA* or *PIK3R1* alterations. (**e**) Pie chart distribution (%) of *PTEN* homozygous CN loss/deletions in molecular subgroups. (**f**) Pie chart distribution (%) of *PTEN* genomic alteration types in the Combined cohort: in-frame, missense mutations; Fs or stop, frameshift or stop/nonsense mutations; deletion, homozygous CN loss. (**g**) Distribution of *PTEN* mutation types in the four largest molecular subgroups. (**h**) Line graph tracing the incidence of the indicated alterations in the five molecular subgroups with n > 10 cases. p53 pathway alterations do not include p14ARF deletions. (**i**) Correlation matrix showing the associations between molecular subgroups and genomic alterations in the indicated cell cycle (CC) G1 phase and p53 pathway effectors. Asterisk (*) indicates statistically significant correlations; *p*-values decrease as the absolute value of the Pearson correlation coefficient increases (see Supplementary Figure S4).

The common genomic alterations in glioblastoma fall into five major pathways driving glioblastoma growth: the canonical ERK/MAPK and PI3K growth pathways, the telomere elongation pathway, the cell cycle G1 phase and the p53 checkpoint pathway (Figure 4a–c and Table 3). the less common mutations were present in DNA damage response and chromatin remodeling pathways, or in transcription factors and effectors from other cell cycle phases (Table 3). The genetic alterations in the two canonical growth pathways were the upstream drivers in most of the glioblastoma cases, with only five G7/Other subgroup cases in the Combined cohort (2.7%) showing no detectable PI3K/PTEN pathway alteration.

Mutations in the three major effectors of the PI3K pathway: *PTEN*, the main tumor suppressor of the pathway; *PIK3CA*, encoding the PI3K catalytic subunit p110α; and *PIK3R1*, encoding the PI3K regulatory subunit p85, and only rare mutations of *MTOR*, were present in three quarters of the glioblastoma cases (Figure 4a and Table 3). Except for rare cases with overlap representing less than 6% of the Combined cohort, the *PTEN* alterations were mutually exclusive with the *PIK3CA* and *PIK3R1* mutations (Figure 4a and Supplementary Figure S3A). From the cases with PI3K alterations, the PTEN alterations occurred usually alone, but more than a quarter of the *PIK3CA* and *PIK3R1* mutant tumors, respectively, also contained another PI3K pathway alteration (Figure 4d). In general, the PI3K alterations displayed relatively similar distributions in the molecular subgroups, except for the G5/PDGFRA and G7/Other subgroups that showed decreased or increased PI3K pathway representation, respectively, mainly due to the incidence of *PTEN* mutations, without statistical significance in the multiple variable correlation matrix analysis (Table 3 and Supplementary Figure S3B). Another parameter enriched in the G7/Other subgroup was the presence of *PTEN* homozygous loss/deletions (Figure 4e). *PTEN* alterations are diverse in glioblastoma and fall into four subgroups: small in-frame alterations, such as missense mutations and few amino acid in-frame deletions, protein truncations by frameshift or stop/nonsense mutations, splice mutations and homozygous gene deletions (Figure 4f). Splice mutations and gene deletions represented approximately one third of the *PTEN* alterations in the whole cohort and over half in the G7/Other subgroup (Figure 4f,g), with more than half of the *PTEN* gene deletions clustered in this subgroup (Figure 4e). Although *PTEN* alterations show deleterious effects through a variety of mechanisms [22,23,24,25] and are accompanied by the loss of heterozygosity usually due to chromosome 10 heterozygous loss, PTEN homozygous deletions are obviously the most severe gene alterations.

As previously reported, the *TERT* promoter mutations showed the lowest incidence in the G5/PDGFRA subgroup [4,26] (Figure 4h). All other subgroups showed an approximate 80% *TERT* promoter mutation rate, except for the G7/Other and especially the G1/EGFR-amplified subgroups, which showed an even higher incidence (Table 3, Figure 4h).

The preferential associations between the gene alterations and molecular subgroups were especially distinct for the effectors of the cell cycle G1 phase and the p53 pathway (Table 3, Figure 4b,c,h,i, Supplementary Figure S4). The alterations of the G1 phase comprised the homozygous loss of *CDKN2A*, usually associated with a neighboring *CDKN2B* locus loss, *CDK4* amplification and *RB1* inactivation due to similar mutational events as shown for *PTEN* in Figure 4f. Similar to the PI3K pathway alterations, the G1 phase alterations were mutually exclusive except for rare cases with overlap, totaling 7.5%, 16.7% and 24.2% of the *CDKN2A*, *CDK4* and *RB1*-mutant cases, respectively, and representing 5.4% of the cases from the Combined cohort (Figure 4b, Supplementary Figure S5A). Although the cases with G1 phase genomic alterations totaled 80–90% in all the molecular subgroups, the distribution of the individual alterations showed subgroup specificity with statistically significant opposite distribution in the G1/EGFR-amplified and G7/Other subgroups (Figure 4i, arrows). As the incidence of *CDKN2A* homozygous loss decreased along the well-represented G1, G3, G5, G6, and G7 subgroups, the incidence of the *CDK4* amplification and *RB1* inactivating mutations variably increased, with an equal number of *CDK4-* and *RB1*-mutant cases in the G6/Multi-RTK and G7/Other subgroups (Figure 4h). The G7/Other subgroup was enriched in cases with *CDK4* or *RB1* alterations (Figure 4b).

As previously noted [4], the *TP53* mutations were inversely correlated with *CDKN2A* homozygous loss (Supplementary Figure S5B) due to simultaneous encoding from the alternate ORFs of p16/INK4A and p14ARF proteins by the *CDKN2A* gene locus. Whereas p16/INK4A inhibits Cyclin D/CDK4-6 complexes, p14ARF leads to the degradation of MDM2 and therefore the stabilization of p53 [27]. *CDKN2A* deletion therefore simultaneously accelerates the cell cycle G1 phase through p16/INK4A downregulation, and upregulates MDM2 to suppress p53 via p14ARF downregulation. Overall, the p53 pathway mutations, mostly contributed by *TP53* mutations (Supplementary Figure S5C) were negatively correlated with *CDKN2A* homozygous deletion and positively correlated with both *CDK4* amplification and *RB1* inactivation (Figure 4h, Supplementary Figure S5B). The strong correlation between *CDK4* and *MDM2* amplifications may be explained by the vicinity of their gene loci on chromosome 12q14.1 and 12q15, respectively (Supplementary Figure S5B). As for the other growth pathways described above, the p53 intrapathway alterations were mutually exclusive, with one exception in a case with simultaneous *MDM2* and *MDM4* amplifications (Figure 4c and Supplementary Figure S5C). The same trend as for the G1 phase effectors opposing the G1/EGFR-amplified and G7/Other subgroups was noted for p53 pathway effectors. *MDM4* amplification showed a striking incidence in the G6/Multi-RTK subgroup (Figure 4c,i).

Besides the genomic alterations in the five major pathways, approximately a third of the tumors showed mutations in the DNA damage response or chromatin remodeling pathways (Table 3). Mutations in other genes were less frequent but they may represent therapeutic targets. For example, inactivating mutations in *STAG2*, a component of the cohesin complex responsible for sister chromatid cohesion following DNA replication, may be targeted by PARP inhibitors [28], and occurred in approximately 10% of the cases, except for those in the G7/Other subgroup.

### 3.5. Implementation into Practice of the G1-G7 Molecular Subgroup Classification of Glioblastoma

A practical tumor molecular classification should be feasible and reliable for the respective type of tumor, and also part of a rapid diagnosis for treatment guidance. Typically, glioblastoma presents as a rim-enhancing mass on magnetic resonance imaging (MRI) and requires histologic pathology diagnosis to differentiate it from a host of other conditions with the same MRI imaging characteristics, including the more frequent metastatic disease. The histologic diagnosis, which consists of the microscopic examination of H&E slides and appropriate IHC stains, is performed routinely in any pathology practice throughout the world and, with very rare exceptions, correctly excludes other possibilities from the differential diagnosis as a first step towards the histologic diagnosis of glioblastoma (i.e., test specificity is nearly 100%). The second step of the histologic diagnosis is establishing that the tumor is a high-grade diffusely infiltrating astrocytoma with either necrosis or microvascular proliferation, which represent the diagnostic criteria for glioblastoma. Although in the large majority of cases, these criteria are met, WHO has recently introduced three molecular pathology criteria to assist with the cases where histology is insufficient, either because the tumor is caught early during progression or because the histologic diagnostic features are not present in the biopsy. These molecular pathology criteria are either the presence of *EGFR* amplification, *TERT* promoter mutation, or combined chromosome 7 gain and 10 loss (7+ and 10−) [2].

**Figure 5:**
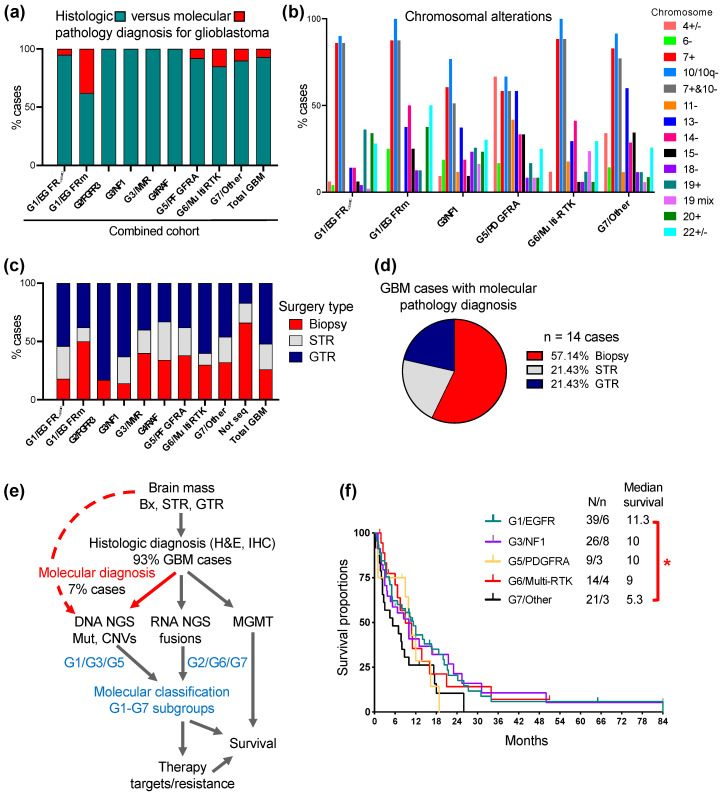
Translation into medical practice of the G1-G7 classification of glioblastoma. (**a**). Proportion (%) of cases diagnosed as glioblastoma by histologic and molecular pathology criteria in the Combined cohort (see also Supplementary Table S1). (**b**). Pertinent chromosomal alterations in selected glioblastoma subgroups (see Supplementary Table S2 for all subgroups). Only entire or almost entire chromosome alterations are shown, except for chromosome 10, for which both entire or q arm losses are shown (10/10q−). Chromosome: +, gain; −, loss; +/−, either gain or loss; 19 mix, 19 with complex losses and gains. Note lower % of cases with combined chromosome 7+ and 10− in G3/NF1 and G5/PDGFRA subgroups. (**c**). Proportion of cases with biopsy, subtotal resection (STR) or gross total resection (GTR) in the Combined cohort. GTR is defined as complete resection of the tumor enhancing area. (**d**). Proportion (%) of biopsies, STRs and GTRs in the 14 glioblastoma cases that required NGS for diagnosis. (**e**). Flowchart depicting the steps for glioblastoma diagnosis and G1-G7 molecular classification. Note that DNA NGS is used both for molecular pathology diagnosis (red labeling) and molecular classification (blue labeling). The molecular subgroups that require only DNA NGS or both DNA NGS and RNA fusion detection are indicated. (**f**). Survival curves for the three major subgroups, and the G5/PDGFRA and G6/Multi-RTK largest minor subgroups. The median survival expressed in months is indicated. N, number of deaths; n, number of patients in active follow-up. Note statistically significant survival difference between G1/EGFR and G7/Other subgroups: *, *p* < 0.05.

The analysis of the combined cohort showed that the histologic diagnosis successfully identified over 90% of the glioblastoma cases (Figure 5a, Supplementary Table S1). Among the molecular subgroups, the G1/EGFR-mutant, G6/Multi-RTK and G7/Other showed the lowest histologic diagnosis rates, with the rest of the cases from these subgroups requiring molecular diagnosis. As a molecular criterion, *EGFR* amplification applies to the cases from the G1/EGFR-amplified subgroup and few cases from the G6/Multi-RTK subgroup (Figure 3c). *TERT* promoter mutation shows very high incidence in glioblastoma but its highest incidence was also detected in the G1/EGFR-amplified subgroup (Table 3). For the third criterion, the analysis of the CNVs in the Combined cohort showed high rates, in the range of 80–90%, of chromosomes 7+ and 10− in all the subgroups, except for the G3/NF1 and G5/PDGFRA subgroups, where it showed 50–60% rates, and the G3/MMR subgroup that lacked it altogether (Figure 5b and Supplementary Table S2). These findings suggest that although the histologic and current molecular criteria cover a large extent of the glioblastoma diagnosis, some cases in subgroups with lower *TERT* promoter mutation and chromosome 7+ and 10− incidence, especially from the G5/PDGFRA subgroup but possibly also from the G3/NF1, G3/MMR, G6/Multi-RTK and G7/Other subgroups, may require additional molecular diagnosis criteria.

The CNV analysis also identified chromosomal alterations enriched in the G1-G7 molecular subgroups (Figure 5b, Supplementary Table S2). The chromosomal alterations highlighted in Supplemental Table S2 are as follows: 19 and 20 gains for the G1/EGFR-amplified subgroup, 6 and 14 losses and 20 gains for the G1/EGFR-mutant subgroup, 18 losses and 19 complex alterations for the G3/NF1 subgroup, 4 alterations and 11 and 15 losses for the G5/PDGFRA subgroup, 14 losses and 19 complex alterations for the G6-Multi-RTK subgroup and chromosome 15 loss for the G7/Other subgroup. Chromosome 13 loss was enriched in the G3/MMR, G5/PDGFRA and G7/Other subgroups, whereas chromosome 22 alterations were less specific and noted in all the subgroups.

Surgical intervention providing viable material suitable for various tests may be limited in a significant number of glioblastoma cases due to factors such as tumor location, extensive tumor necrosis and patient co-morbidities. In the Combined cohort, biopsies with limited material were obtained in over one quarter of the total cases but with a much higher incidence in some molecular subgroups, such as the G1/EGFR-mutant (Figure 5c, Supplementary Table S3). Not surprisingly, four out of six cases without molecular results (labeled “Not seq”) were biopsies, and a fifth, a subtotal resection with limited material, all failed NGS, whereas the sixth case was a gross total resection with NGS cancelled due to the patient’s demise. The assessment of the cases requiring molecular pathology diagnosis showed also a predominance of biopsies (Figure 5d), furthermore stressing the importance of using a highly sensitive, specific and tissue-sparing molecular approach with multiple purpose usage, such as the NGS.

The flowchart streaming the diagnostic and G1-G7 molecular classification approach for glioblastoma is presented in Figure 4e. The first obligatory step due to many considerations, some of them mentioned above, is the histologic diagnosis. The next step is the prioritized submission of tissue to DNA NGS for mutation and CNV detection, a step necessary and sufficient for both the pathology molecular diagnosis and for the classification in the G1/EGFR, G3/NF1, G3/MMR and G5/PDGFRA subgroups. The RNA NGS is performed in many laboratories in the same time as the DNA NGS, by dual DNA and RNA extraction from FFPE samples, and this technique is preferred and recommended for all cases, as it covers also scant tissue samples. If RNA NGS is not available, RNA fusion detection as a distinct approach should be attempted at least for the cases that did not fall in one of the four subgroups mentioned above, although rare cases with RTK heterogeneity by gene amplification and fusion, such as the G6/Multi-RTK case #6 (Figure 3), may be missed. RNA fusion detection is necessary for classification into the G2/FGFR3, G4/RAF and G6/Multi-RTK subgroups that contain many cases with gene fusions targetable by clinically available inhibitors. By exclusion, the G7/Other cases do not contain fusions. MGMT promoter methylation testing, usually performed separately from NGS, is usually also required by the oncologist for predicting temozolomide response.

The G1-G7 classification allows for the selection of cases for targeted treatment (see Discussion) and may also show prognostic significance even in the context of non-personalized therapy, which is the current mainstay for glioblastoma. In the Combined cohort, the therapeutic options for glioblastoma revolved around surgery (Figure 5c and Supplementary Table S3), and adjuvant radiation and temozolomide in the large majority of patients, with bevacizumab being administered in selected cases at recurrence. Rare patients with severe deterioration opted only for end-of-life supportive measures after surgery. The preliminary survival curves showed a shorter median survival for the G7/Other subgroup, with a statistically significant difference versus the G1/EGFR-amplified subgroup (Figure 5f and Table 3). The median survival is detailed in Table 3 for all the G1-G7 subgroups, showing at this stage only preliminary values for the minor subgroups without a sufficient number of cases for definitive analysis. Noteworthily, the G1/EGFR and G5/PDGFRA subgroups showed similar median survivals as previously reported for the Discovery cohort [4], whereas the G3/NF1 and G6/Multi-RTK subgroups with high accrual in the Validation cohort showed an increase in the median survival to 10 and 9 months, respectively. 

## 4. Discussion

**Figure 6:**
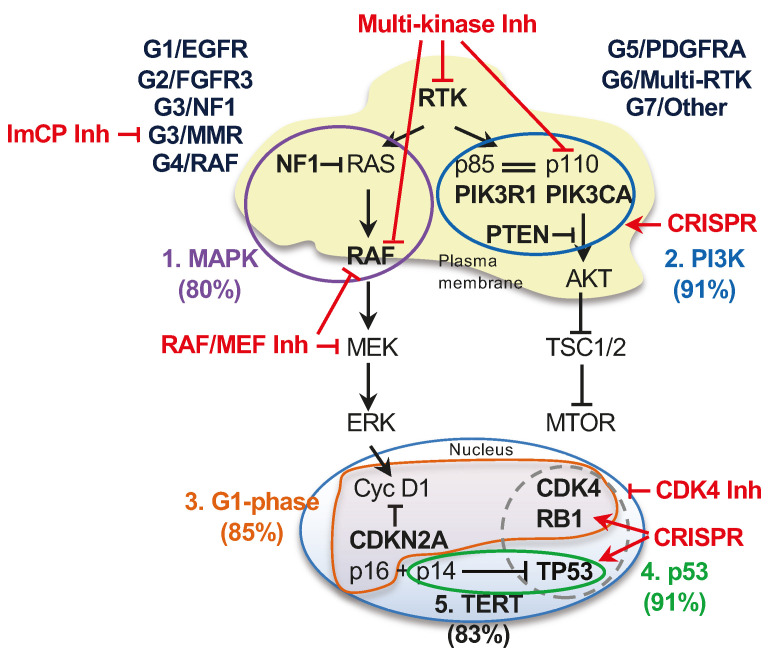
Molecular subgroups, major growth pathways and proposed therapeutic approaches in glioblastoma. Schematic diagram of the five major growth pathways driving glioblastoma in the G1-G7 molecular subgroups. The genes most commonly altered in a pathway are indicated in bold; the RTKs showing frequent genomic alterations are *EGFR*, *PDGFRA*, *MET* and *FGFR3*, each included in a molecular subgroup. The pathways are numbered and color-coded and the approximate % case involvement is indicated, as calculated from n = 186 glioblastoma cases with successful NGS. RTK alterations are included in both canonical growth pathways, whereas the p14ARF alternate ORF of the *CDKN2A* gene is included in the p53 pathway in this diagram. The cellular subcompartmentalization is also represented, illustrating that the majority of genomic alterations target effectors localized either at the plasma membrane for the two canonical growth pathways or in the nucleus for TERT, cell cycle G1 phase and p53 pathways. The most frequently altered pathway effectors are included in color-coded pathway encircling. The interaction between p85 and p110 subunits of the PI3K is indicated by double line. The frequent association of genomic alterations in *TP53* and *CDK4* or *RB1* is encircled by dashed line. Proposed pharmacologic interventions are labeled in red: Inh, pharmacological inhibitor; ImCP, immune checkpoint.

The molecular classification of neoplasms, including CNS tumors, has provided a better understanding of their pathogenesis, progression and also molecular targets for therapy. Glioblastoma has been one of the few CNS tumors without a comprehensive and all-inclusive molecular classification until two years ago, when I proposed the MAPK pathway-based G1-G7 molecular classification [4]. The current study validates this classification by showing that all the cases from the Validation cohort prospectively mapped into the G1-G7 molecular subgroups defined in the Discovery cohort. The classification was further refined by separating the rare glioblastoma cases with MMR deficiency into a G3/MMR molecular subgroup, a spin-off of the G3/NF1 subgroup, since RAS activation appears to be dominant in the MMR tumors, as well. The patients from this subgroup may benefit from immune checkpoint inhibitors, similarly to pediatric patients with glioblastoma in the context of MMR deficiency [29,30], while patients from G3/NF1 and G4/RAF subgroups may respond to targeted therapy with RAF and MEK inhibitors [31] (Figure 6). Noteworthily, none of these clinical trials showed responses in unclassified cohorts of glioblastoma [31], emphasizing the need for rational patient trial enrollment based on the underlying molecular mechanisms operating in glioblastoma subgroups.

The Validation cohort case accrual rate per subgroup showed important differences relative to that of the Discovery cohort, resulting in a balanced molecular subgroup distribution in the three major subgroups and a significantly enlarged G6/Multi-RTK subgroup. Since race has been shown to contribute to molecular differences between glioblastoma cohorts [32], I examined cohort demographic disparities and found age as the main significantly different parameter, the Validation cohort showing a much higher median age than the Discovery cohort. Coupled to the observation that age was significantly higher for the G7/Other and G6/Multi-RTK groups than for the G1/EGFR subgroup patients, the age difference between the cohorts reflected the opposite accrual trends and resulted in a significant relative increase in the G7/Other and G6/Multi-RTK cases in the Validation cohort. Most likely, age, coupled to a higher number of female patients contributing tumors to the G3/NF1 subgroup, explains the balanced distribution of the major subgroups in the Validation cohort, and suggests age and sex dependence for glioblastoma molecular profiles.

In contrast, the racial composition was similar in the two cohorts, with an almost 5:1 C/W to AA/B ratio. Since no molecular analysis was carried out in the literature for the AA/B population in glioblastoma, the comparison of demographic and molecular parameters between the C/W and AA/B cases became extremely important. This preliminary analysis showed a significantly higher TMB in WHO grade 4 gliomas from AA/B patients, including glioblastomas, although not reaching the level set currently for treatment with immune checkpoint inhibitors for other solid cancers [33]. Another observation in AA/B patients was the younger onset age for WHO grade 4 gliomas, in general. The subgroup distribution of the AA/B tumors showed a balanced distribution of all the major subgroups, as well as the largest minor subgroups G5/PDGFRA and G6/Multi-RTK. The only major differences concerned the expanded G1/EGFR-mutant and G3/NF1 subgroups, the former most likely because of a disproportionately high male to female 9:1 ratio in the G1/EGFR subgroup. These preliminary observations require confirmation in a higher number of cases.

Driver genetic alterations in cancer fall into three major categories: mutations, CNVs and gene fusions. In glioblastoma, most RTK driver alterations were CN gains/amplifications, accounted for by *EGFR*, *PDGFRA*, and to a lesser extent, *MET*. Approximately 10% of the G1/EGFR and G5/PDGFRA cases showed only RTK mutation in the absence of amplification. The amplified RTK may or may not show simultaneous mutations, usually of the extracellular domain, splice variants rendering the RTK constitutively active (e.g., EGFR vIII), and fusions (e.g., EGFR-SEPT14) [4,34]. The novel *ST7–MET* fusion described in this study falls also into this category of fusions of amplified RTKs. However, RTK fusions may occur in the absence of RTK amplification, such as the FGFR3 fusions that show only a 3-fold increase in *FGFR3* expression [7], or *PTPRZ1–MET*, *LMNA–NTRK1* and *GOPC1–ROS1* fusions that showed over 15-fold RNA expression increases due to transcription from the fusion partner promoter. Whereas the mechanism of constitutive activation has been studied for the rare *GOPC1–ROS1* fusion and includes changes in subcellular localization [21] in addition to the overexpression driven by the GOPC1 promoter shown in this study, the activation of *PTPRZ1–MET* fusions is yet to be studied, including the putative involvement of mechanisms other than overexpression [35]. Although these fusions are rare in glioblastoma, the incidence in the Combined cohort being 0.5% for *ST7–MET* and *LMNA–NTRK1*, 1.1% for *PTPRZ1–MET* and *GOPC–ROS1*, and 1.6% for *FGFR3* fusions [7], they appear to be targetable alterations that also occur in other solid cancers [36]. Currently, the only FDA-approved personalized treatment for glioblastoma is with Entrectinib/Larotrectinib for tumors with an *LMNA–NTRK1* fusion [37].

This study underscores an important finding relating the signaling through the MAPK with three other major pathways activated in glioblastomas, the PI3K, cell cycle G1 phase and p53 pathways (Figure 6). Because the high number of cases in the Combined cohort allowed for powerful statistical correlations, a multivariate analysis found significant intra- and inter-pathway associations. Most of these specificities were suggested as trends from the analysis of the Discovery cohort, warranting the hierarchic clustering of the G1-G4 subgroups in molecular cluster #1, of the G6-G7 subgroups in molecular cluster #2 and of the G5/PDGFRA subgroup as stand alone, mostly due to the lower incidence of *TERT* promoter mutations in this latter subgroup [4]. In general, the molecular cluster #1 subgroup tumors rely on G1 phase activation through *CDKN2A* homozygous loss, whereas, increasingly, the G5 to G7 subgroup tumors rely on *CDK4* and *RB1* alterations in combination with p53 pathway alterations (Figure 6, see also Figure 4h,i). The multivariate matrix analysis also found opposing contributions of the G1 phase and p53 pathway effectors to the G1/EGFR and G7/Other subgroups, further supporting the distinction between these two major subgroups that is also reflected in a statistically significant survival difference. In addition, the refinement of the types of PTEN mutations showed the enrichment of PTEN homozygous deletions in the G7/Other subgroup, indicating the strong dependence of these tumors on PI3K signaling.

Previously, single RTK therapeutic targeting strategies in glioblastoma have been unsuccessful due to a variety of drug resistance mechanisms, including cancer cell reprogramming and tumor heterogeneity [9,38,39,40]. The G6/Multi-RTK subgroup assembles tumors showing RTK genomic heterogeneity, but a multitude of RTKs without genomic alterations are overexpressed in glioblastoma, such as *KDR*, *MET*, *NTRK1*, *ALK*, *ERBB2*, *EPHB1-4*, *EPHA3*, *PTK7* and *ROR1-2*, with or without preferential expression in the G1-G7 molecular subgroups [4]. Therefore, approaches targeting multiple RTKs, such as using multi-kinase inhibitors, especially in the G6/Multi-RTK subgroup, appear to be a more rational approach (Figure 6). Indeed, regorafenib, a multi-kinase inhibitor [41], has shown promise in recurrent glioblastoma [42]. The genomic heterogeneity extended also to the PI3K and G1 cell cycle phase effectors, with 5–6% of cases showing mutational overlap for each pathway in the Combined cohort. Multifocal molecular analyses have shown spatial heterogeneity for multiple RTKs and PI3K effectors [7,9,15], and these comprehensive autopsy-based genomic–proteomic studies are invaluable for understanding the complex signaling in glioblastoma.

PTEN mutations, and more generally, activating mutations of the PI3K pathway, have been linked to resistance to RTK therapy [43,44] (Figure 6). Unfortunately, PI3K pathway inhibitors have not shown efficacy due to releasing negative feedback loops that re-activate growth signaling through RTKs [5]. Novel, more physiologic approaches, such as re-activating PTEN tumor suppressor by repairing the point mutations through CRISPR gene editing [45] or suppressor tRNA [46] might more efficiently lead to tumor cell death (Figure 6). Of note is that these editing technologies may be applied to both tumor suppressors that are inactivated mostly by mutations and not deletions, such as *PTEN*, *TP53*, *RB1* and *NF1*, and to oncogenes, such as *PIK3CA* [45].

## 5. Conclusions

In conclusion, the G1-G7 molecular subgroup classification of glioblastomas represents the first validated all-inclusive molecular classification of glioblastoma. All prospective glioblastoma cases, including those driven by novel alterations, such as *ST7–MET* fusion or *PDGFRB* amplification, were successfully assigned to one of the subgroups. Demographic factors, such as age, sex and race, were shown to enrich certain subgroups. Besides the upstream RTK and MAPK pathway driver molecular alterations, the associations with the major growth pathways rendered specificity to the subgroups. The practicality of combining the G1-G7 classification with other necessary steps for diagnosis and target identification in small tissue samples is a net advantage as compared to approaches based on RNA expression or CNVs, which are suitable for other CNS tumors [2]. The G1-G7 classification will thus become critical for patient enrolment in clinical trials, as positive responses are more likely to be obtained by specifically targeting tumors with similar molecular characteristics, i.e., from a defined molecular subgroup, than by a general therapeutic approach to all tumors. Future developments also aim to include transcriptomic and proteomic approaches for detection and monitoring pathway activation in order to integrate the molecular evolution triggered by responses to therapy.

## Figures and Tables

**Table 1:** Demographic diversity in glioblastoma cohorts.

GBM Cohorts	Age ^1^	Age M ^1^	Age F ^1^	Age W ^1,2^	Age B ^1,2^	M:F Ratio	W:B Ratio ^2^	W M:F Ratio	B M:F Ratio
Combined n = 192	65	64.5	68	66	62	1.46:1	4.7:1	1.4:1	1.75:1
Discovery n = 89	62	62	63	62	63	1.5:1	4.9:1	1.4:1	4:1
Validation n = 103	70	70.5	70	72	61	1.4:1	4.6:1	1.5:1	1:1

GBM, glioblastoma; M, male; F, female; W, Caucasian/White; B, African American/Black. ^1^ Median age expressed in years. ^2^ Race demographic analyses do not include two Hispanic and one Asian patients.

**Table 2:** Demographic characteristics of glioblastoma molecular subgroups.

GBM Subgroups	Cases	Age ^2^	M:F Ratio	W:B ^3^ Ratio	W M:F Ratio	B M:F Ratio	TMB ^4^
G1/EGFR	59	63 (50)	2.1:1	4.9:1	1.7:1	9:1	4.5 ± 0.2
G2/FGFR3	5	62 (16)	1:4	W only	1:4	NA	4.5 ± 1.1
G3/NF1	43	65 (61)	1.1:1	3.3:1	1.1:1	1.5:1	4.7 ± 0.2
G3/MMR	4	71 (17)	3:1	W only	3:1	NA	23 ± 6.4
G4/RAF	6	63 (51)	1:2	W only	1:2	NA	4.3 ± 0.9
G5/PDGFRA	13	68 (44)	2.2:1	5:1	4:1	1:1	5.0 ± 0.7
G6/Multi-RTK	18	69 (35)	1.6:1	5:1	1.5:1	2:1	4.4 ± 0.5
G7/Other	38	72 (55)	1.4:1	4.3:1	1.5:1	0.75:1	3.7 ± 0.3
GBM total sequenced ^1^	186	65 (66)	1.5:1	4.7:1	1.4:1	1.9:1	4.4 ± 0.1

GBM, glioblastoma; N, number; M, male; F, female; W, Caucasian/White; B, African American/Black; TMB, tumor mutation burden; NA, not applicable. ^1^ Six GBMs failed sequencing: 3 WM, 2 WF and 1 BF. ^2^ Median age with range in parenthesis, expressed in years. ^3^ Race analyses do not include one Asian and two Hispanic patients (2M and 1F), classified as G7/Other, G4/RAF and G5/PDGFRA, respectively. ^4^ TMB (mutations/DNA kbase) expressed as mean±SEM (standard error of the mean). Total GBM TMB excludes the G3/MMR cases.

**Table 3:** Mutation % frequency for major pathways, and survival in glioblastoma subgroups ^1^.

Gene	GBM n = 186	G1/EGFR ↑ n = 52	G1/EGFRm n = 7	G2/FGFR3 n = 5	G3/NF1 n = 43	G4/RAF n = 6	G5/PDGFRA n = 13	G6/Multi-RTK n = 18	G7/Other n = 38
TERT	83.1	95.9	85.7	80.0	79.1	83.3	61.5	83.3	86.8
PTEN	55.9	51.9	57.1	80.0	55.8	66.7	30.8	61.1	60.5
PIK3CA	16.1	17.3	0	0	16.3	0	23.1	11.1	15.8
PIK3R1	7.5	7.7	14.3	0	11.6	0	7.7	5.6	5.3
PI3K/mTOR ^2^	75.3	75.0	57.1	80.0	76.7	66.7	53.8	72.2	84.2
CDKN2A↓	57.8	76.9 *	42.9	80.0	69.8	80	61.5	55.6	21.0 *
CDK4↑ ^3^	13.0	5.8 *	14.3	0	0 *	0	30.8	16.7	34.2 *
RB1	17.8	7.7	42.9	20.0	18.6	0	0	16.7	34.2 *
G1 phase ^4^	84.9	86.5	100	100	83.7	80.0	92.3	88.9	78.9
TP53	34.4	17.3 *	57.1	20.0	30.2	16.7	30.8	50.0	50.0 *
MDM2↑	5.9	1.9	0	20.0	2.3	0	15.4	5.6	13.2 *
MDM4↑	8.6	11.5	0	0	2.3	0	15.4	27.8 *	7.9
RPL5	5.4	5.8	0	0	7.0	16.7	0	5.6	2.6
TP53 path ^5^	54.8	36.5 *	57.1	40.0	41.9	33.3	69.2	83.3 *	76.3 *
DDR path ^6^	28.0	26.9	14.3	60	27.9	16.7	46.1	22.2	18.4
SWI/SNF ^7^	10.2	11.5	0	0	16.3	16.7	7.7	11.1	0
Other ChRm ^8^	23.1	21.1	0	80	20.9	0	23.1	27.8	21.0
STAG2	9.1	13.5	28.6	20	9.3	16.7	7.7	5.6	0
Survival ^9^	9	11.3	13	20	10	5.3	10	9	5.3

↑, gene amplification; ↓, homozygous CN loss; *, statistical significance (see correlations matrices for Pearson’s correlation coefficients); m, point mutation; path, pathway; DDR, DNA damage response; ChRm, chromatin remodeling. ^1^ The G3/MMR is not shown separately due to low number of cases. ^2^ % cases with at least one alteration in *PTEN*, *PIK3CA*, *PIK3R1* or *MTOR*. ^3^ *CDK4*↑ in all molecular subgroups, except the G6/Multi-RTK, in which two cases show *CDK6*↑, either alone or in combination with *CDKN2A*↓. ^4^ % cases with *CDKN2A*, *CDK4*/*6* or *RB1* alterations. ^5^ % cases with *TP53*, *MDM2*, *MDM4*, *RPL5* and *PPM1D* or *XPO1* alterations, the latter two with each one altered in the G5/PDGFRA and G7/Other subgroups, respectively. ^6^ % cases with either MMR (*MSH2*, *MSH5*, *MSH6*, *PMS2*, *MLH1*, *MLH3*, *MUTYH*, *POLE*) pathogenic mutations or in various genes involved in DNA repair by homologous recombination (*ATM*, *ATR*, *BRCA2*, *BRCA1*, *BRIP1*, *GEN1*, *CHD4*, *MRE11*, *NBN*, *RECQL4*, *RAD51*, *ERCC3*, *ERCC5*, *FANCI*) or nonhomologous end joining (*PRKDC*, *ASTE1*, *CHD2*, *SHPRH*, *PARP2*). ^7^ % cases with SWI/SNF complex *ARID1A*, *ARID1B*, *ARID2*, *SMARCA1*, *SMARCA4*, *SMARCD1* or *PBRM1* pathogenic mutations. ^8^ % cases with either *YEATS4* amplification or *DNM3TA*, *TET2*, *EP300*, *KDM5C*, *KDM6A*, *KMT2C*, *KMT2D*, *NPM* or *CREBBP* pathogenic mutations. ^9^ Median survival measured in months; the number of patients is indicated in Figure 5f, except for the G1/EGFRm (N/n = 6/1), G2/FGFR3 (N = 5) and G4/RAF (N = 5).

## Data Availability

The data presented in this study are available upon request from the corresponding author.

## Supplementary Materials

The following supporting information can be downloaded at https://www.mdpi.com/article/10.3390/cancers16020361/s1, Figure S1: Glioblastoma cohorts: age and race comparison; Figure S2: G6/Multi-RTK subgroup: tumor morphology; Figure S3: PI3K pathway genomic alterations: intrapathway and molecular subgroup correlations; Figure S4: Subgroup associations with G1-phase and p53 pathways effectors: correlation matrix p values; Figure S5: G1 cell cycle phase and p53 pathway genomic alterations: intrapathway and interpathway correlations; Table S1: Proportion of cases diagnosed by histologic or molecular criteria in the G1-G7 subgroups; Table S2: CMV analysis: chromosome alterations in G1-G7 molecular subgroups; Table S3: Surgical modalities in the G1-G7 molecular subgroups.
