# Supplementary material for: Translation into Clinical Practice of the G1-G7 Molecular Subgroup Classification of Glioblastoma: Comprehensive Demographic and Molecular Pathway Profiling
Source: Cancers (Basel). 2024 Jan 15;16(2):361. doi: 10.3390/cancers16020361 (PMC10814912; doi:10.3390/cancers16020361)
Supplement: Supplementary file 1 [file cancers-16-00361-s001.zip › cancers-2759359-supplementary.pdf]

SUPPLEMENTAL FIGURES S1-S5 AND TABLES S1-S3

FIGURES

S1: Glioblastoma cohorts: age and race comparison.
S2: G6/Multi-RTK subgroup: tumor morphology.
S3: PI3K pathway genomic alterations: intrapathway and molecular subgroup correlations.
S4: Subgroup associations with G1-phase and p53 pathway effectors: correlation matrix p values.
S5: Cell cycle G1 phase and p53 pathway genomic alterations: intrapathway and interpathway correlations.

TABLES

S1: Proportion of cases diagnosed by histologic or molecular criteria in the G1-G7 subgroups.
S2: CMV analysis: Chromosome alterations in G1-G7 molecular subgroups.
S3: Surgical modalities in the G1-G7 molecular subgroups.

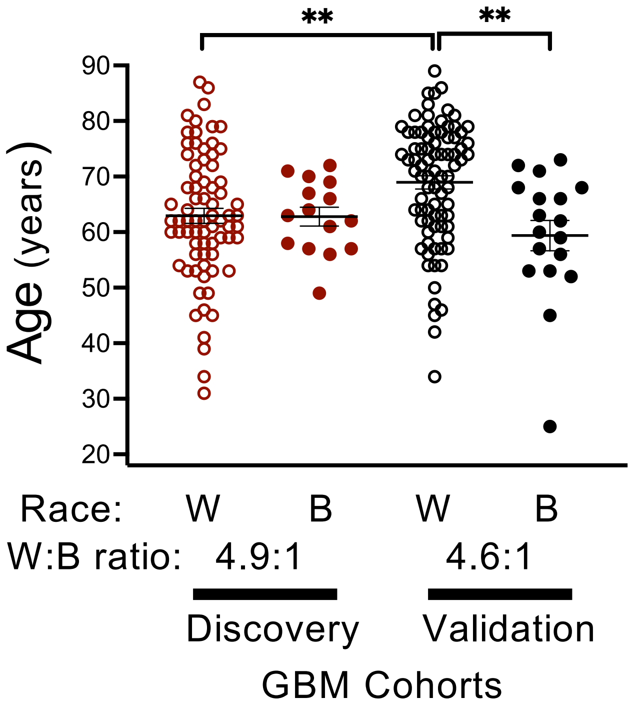

Supplemental Figure S1*.* **<u>Glioblastoma cohorts: age and race comparison.</u>** Age distribution as mean±SEM.of individual values in Caucasian/White (W) and African-American/Black (B) patients from the Discovery and Validation glioblastoma (GBM) cohorts. Statistical significance: **, $p<0.005$.

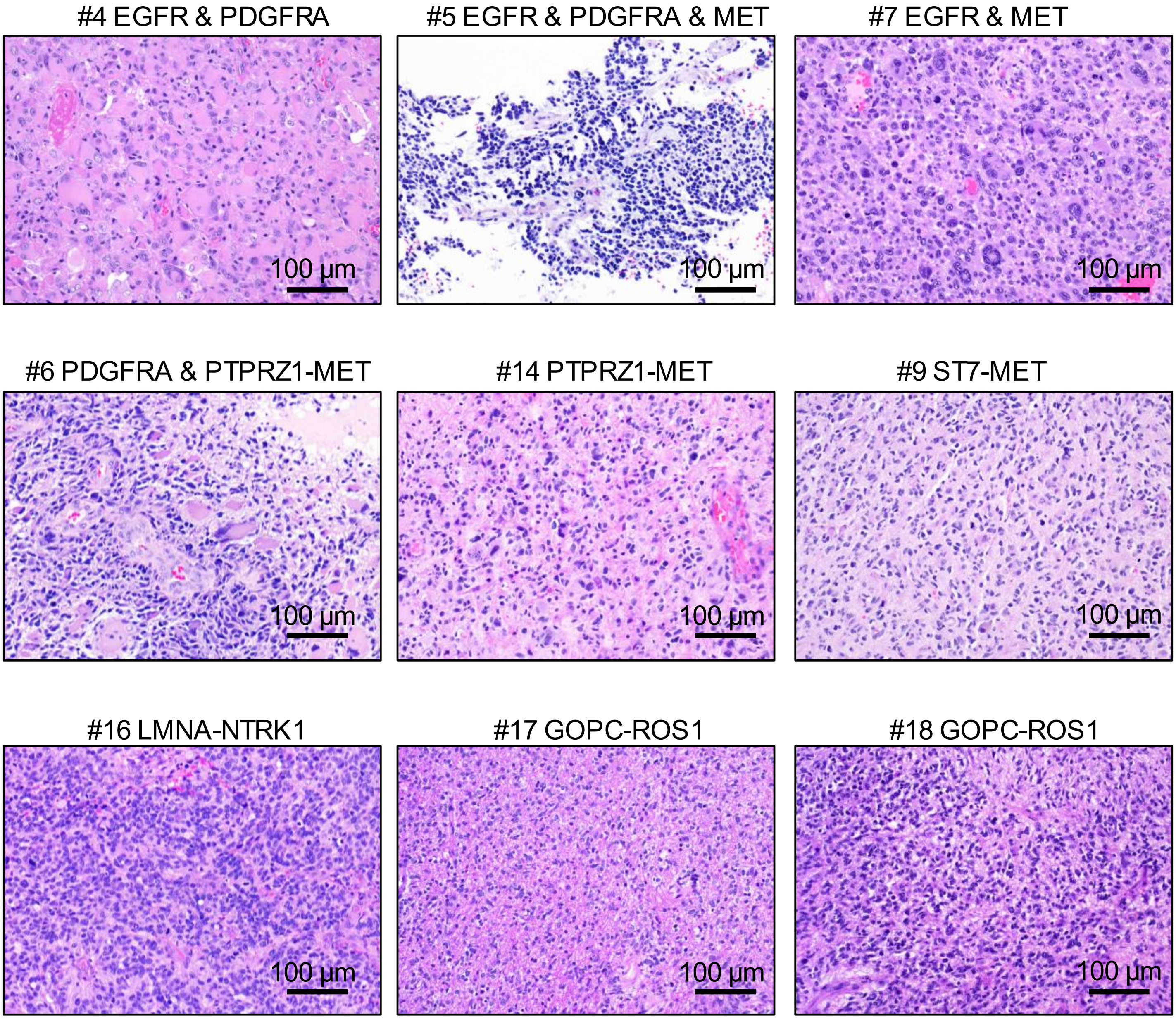

Supplemental Figure S2*.* **<u>G6/Multi-RTK subgroup: tumor morphology.</u>** The morphology in the G6/Multi-RTK subgroup varies along histologic patterns belonging to the #3/Anaplastic and #5/Epithelioid clusters, the latter mostly with gemistocytic and giant cell morphologies. In the #3/Anaplatic cluster, most cases showed pre-HGNE (high-grade neuroendocrine) morphology and only two cases, #5 and #6, both with *PDGFRA* and *MET* alterations, showed HGNE morphology.

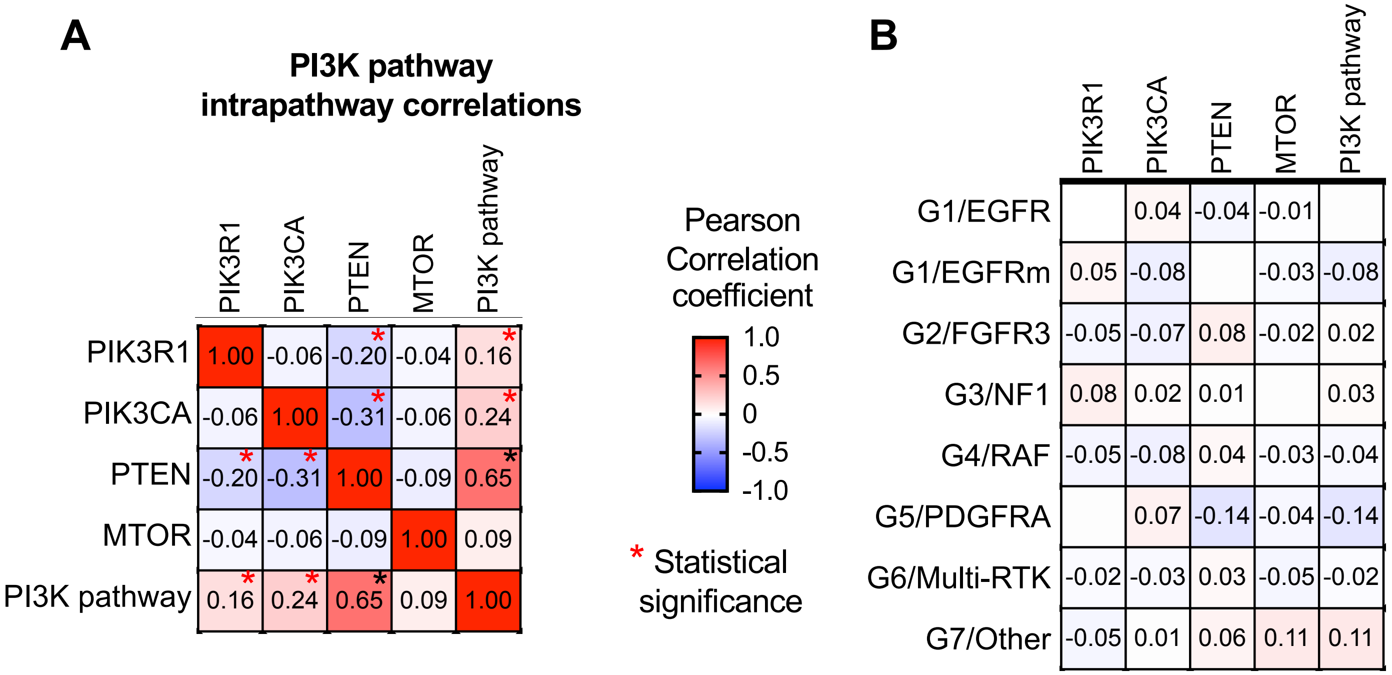

Supplemental Figure S3. <u>PI3K pathway genomic alterations: intrapathway and molecular subgroup correlations</u>.

A. Correlation matrix showing PI3K intrapathway associations between its effectors. Note statistically significant inverse correlations between *PTEN* and either *PIK3R1* or *PIK3CA* alterations. Note also significant contributions of *PTEN* genomic alterations, accounting for most of the cases, *PIK3CA* and *PIK3R1* mutations to the PI3K pathway mutational landscape of the entire glioblastoma cohort. Asterisk (*) indicates statistically significant correlations; p-values decrease as the absolute value of the Pearson correlation coefficient increases.

B. Correlation matrix showing the associations between the mutant PI3K effectors and the molecular subgroups. Note that there is negative correlation between PI3K pathway mutations (absence of PTEN mutations) and the G5/PDGFRA molecular subgroup not reaching statistical significance.

	CDKN2A/2B	CDK4/6	RB1	CC G1 phase	TP53	MDM2	MDM4	RPL5	p53 pathway
G1/EGFRa	0.00123498	0.02908547	0.04658202	0.63488473	0.00130409	0.08128105	0.66780354	0.76717243	0.00029289
G1/EGFRm	0.57460197	0.92431026	0.12084127	0.24344756	0.28258094	0.44531871	0.36237370	0.52993044	0.84949212
G2/FGFR3	0.72167991	0.82721764	0.97359421	0.31517780	0.40328734	0.19166302	0.43276816	0.58848356	0.87014213
G3/NF1	0.09027526	0.00169618	0.68249627	0.86626711	0.98408226	0.16902376	0.06168429	0.47119463	0.12856411
G4/RAF	0.72167991	0.32542199	0.25963459	0.17237123	0.40328734	0.51092976	0.43276816	0.15100215	0.32194673
G5/PDGFRA	0.46646433	0.06308037	0.09029781	0.49245532	0.89182420	0.18859057	0.42113779	0.41692306	0.23567437
G6/Multi-RTK	0.66060509	0.85225346	0.78722583	0.90805937	0.07840290	0.76267177	0.00922890	0.92282546	0.01141967
G7/Other	0.00000001	0.00000083	0.00541539	0.27001009	0.01215739	0.01979488	0.87791524	0.46558644	0.00031069

Supplemental Figure S4. <u>Subgroup associations with G1-phase and p53 pathway effectors: p values for the correlation matrix showed in Fig. 4I.</u> The p values for the correlation matrix in Fig. 4I showing the associations between the G1-G7 molecular subgroups and the cell cycle (CC) G1-phase and p53 pathway effectors are illustrated color-coded for direct correlations (brown hues) or inverse correlations (blue hues).

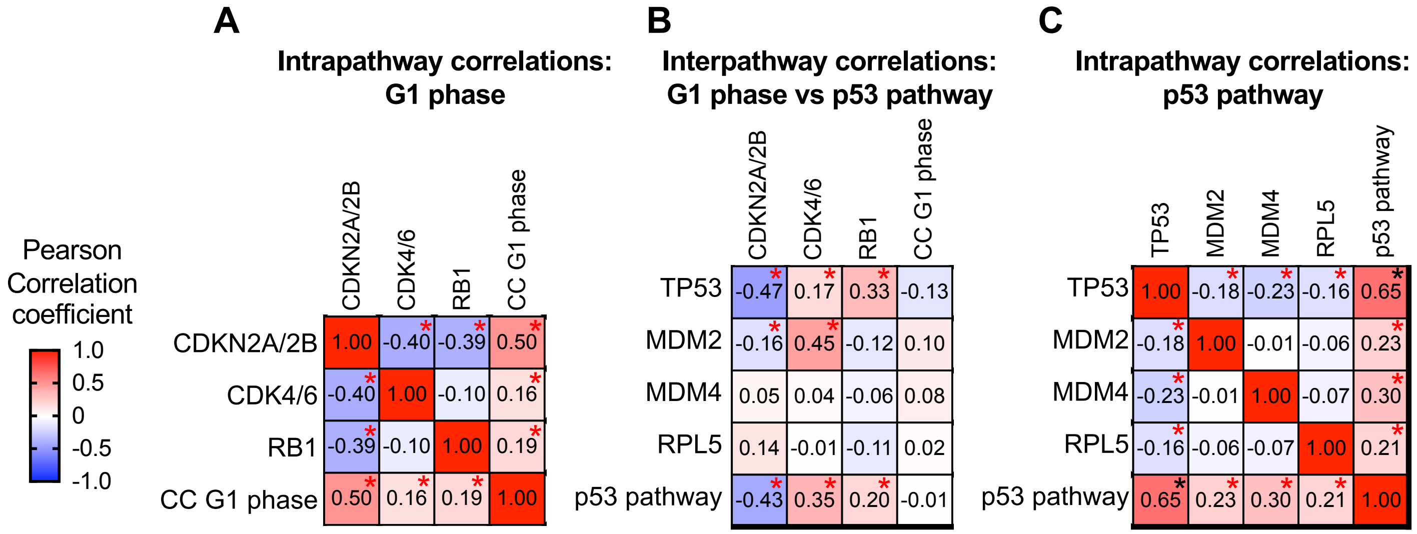

Supplemental Figure S5. __**Cell cycle G1 phase and p53 pathway genomic alterations: intrapathway and interpathway correlations.**__

A. Correlation matrix showing cell cycle (CC) G1 phase intrapathway associations between its effectors. Note statistically significant inverse correlations between *CDKN2A/2B* homozygous loss and either *CDK4* or *RB1* alterations. The two cases with *CDK6* amplification from the G6/Multi-RTK subgroup were also included. Note also significant contributions of *CDKN2A/2B* deletions, accounting for most of the cases, *RB1* and *CDK4/6* alterations to the G1 phase mutational landscape of the entire glioblastoma cohort. Asterisk (*) indicates statistically significant correlations; p-values decrease as the absolute value of the Pearson correlation coefficient increases.

B. Interpathway correlation matrix showing the associations between the alterations of G1 phase and p53 pathway effectors. Note strong inverse correlation between *CDKN2A/2B* deletions and *TP53* mutations, and less with MDM2 amplification, strong direct correlation between *CDK4/6* amplification and *MDM2* amplification, and less with *TP53* mutations, and strong direct correlation between *RB1* and *TP53* alterations. *RPL5* mutations appear to associate with *CDKN2A/B* deletion, but more cases are necessary for statistical significance.

C. Correlation matrix showing p53 intrapathway associations between its effectors. Note statistically significant inverse correlations between *TP53* alterations and either *MDM2, MDM4* or *RPL5* alterations. Note also the most important contribution of *TP53* to the p53 pathway mutational landscape, followed by *MDM4*, *MDM2* and *RPL5*.

Supplemental Table S1: Histologic versus molecular criteria for glioblastoma (GBM) diagnosis in the Combined cohort.

GBM Subgroup	*Histology (% cases)	^Molecular (% cases)
G1/EGFR-amplified	95	5
G1/EGFR-mutant	62	38
G2/FGFR3	100	0
G3/NF1	100	0
G3/MMR	100	0
G4/RAF	100	0
G5/PFGFRA	92	8
G6/MultiRTK	85	15
G7/Other	90	10
Total GBM	93	7

* Histologic criteria include necrosis and/or microvascular proliferation.
^ Molecular criteria are EGFR amplification, TERT promoter mutation or chromosome 7 gain&10 loss in an IDH-wild-type diffuse astrocytoma.

Supplemental Table S2: Proportions (%) of glioblastoma (GBM) cases with indicated entire* chromosome alterations.

*Only entire or almost entire chromosome alterations are shown, except for chromosome 10, where either entire or q arm losses are included (10/10q-).
Only the chromosomes with high incidence of alterations in glioblastoma are shown.

N, number of cases; Chromosome: +, gain; -, loss; +/-, either gain or loss;
19 mix, chromosome 19 with complex losses and gains.

Chromosomes		**4+/-**	**6-**	**7+**	**10/10q-**	**7+&10-**	**11-**	**13-**	**14-**	**15-**	**18-**
**Subgroups**	**N**										
G1/EGFRa	50	6	4	86	90	86	0	14	14	6	4
G1/EGFRm	8	0	**25**	87.5	100	87.5	0	37.5	**50**	25	12.5
G2/FGFR3	6	16.7	16.67	100	100	100	0	16.67	0	0	0
G3/NF1	43	9.3	18.6	60.5	76.7	**51.2**	11.6	37.2	18.6	9.3	**23.3**
G3/MMR	5	20	0	40	20	0	0	**60**	20	0	20
G4/RAF	4	0	0	100	100	100	0	0	25	0	0
G5/PFGFRA	12	**66.7**	16.7	58.3	66.7	**58.3**	**41.7**	**58.3**	33.3	**33.3**	8.3
G6/MultiRTK	17	11.8	0.0	88.2	100.0	88.2	17.6	29.4	**41.2**	5.9	5.9
G7/Other	35	14.3	14.3	82.9	91.4	77.1	11.4	**60.0**	28.6	**34.3**	11.4

Chromosomes	**19+**	**19 mix**	**20+**	**22+/-**
**Subgroups**				
G1/EGFRa	**36**	2	**34**	28
G1/EGFRm	12.5	0	**37.5**	50
G2/FGFR3	**33.3**	0.0	16.7	33.3
G3/NF1	25.6	**16.3**	23.3	30.2
G3/MMR	20	0	20	40
G4/RAF	25	25	50	75
G5/PFGFRA	16.7	8.3	8.3	25.0
G6/MultiRTK	11.8	**23.5**	5.9	29.4
G7/Other	11.4	5.7	8.6	25.7

Supplemental Table S3: Proportions (%) of glioblastoma (GBM) cases with biopsy, subtotal resection (STR) or gross total resection (GTR) in the Combined cohort.

Molecular Subgroups	Biopsy	STR	GTR
G1/EGFR-amplified	18	28	54
G1/EGFR-mutant	50	12	38
G2/FGFR3	17	0	83
G3/NF1	14	23	63
G3/MMR	40	20	40
G4/RAF	34	33	33
G5/PFGFRA	38	24	38
G6/MultiRTK	30	10	60
G7/Other	32	22	46
Not sequenced	66	17	17
Total GBM	26	22	52

Total resection for glioblastoma refers only to the enhancing area on brain imaging.
